# Editorial: The Interplay Between Long-Term Psychiatric Disorders and Age-Related Brain Changes

**DOI:** 10.3389/fpsyt.2022.898023

**Published:** 2022-04-29

**Authors:** Gilberto Sousa Alves, Sanjeev Kumar, Felipe Kenji Sudo

**Affiliations:** ^1^Post-Graduation in Psychiatry and Mental Health (PROPSAM), Federal University of Rio de Janeiro, Rio de Janeiro, Brazil; ^2^Translational Psychiatry Research Group, Federal University of Maranhão, São Luís, Brazil; ^3^Centre for Addiction and Mental Health, Toronto, ON, Canada; ^4^Temerty Faculty of Medicine, University of Toronto, Toronto, ON, Canada; ^5^D'Or Institute for Research and Education (IDOR), Rio de Janeiro, Brazil

**Keywords:** cognition, psychiatric disorders, cognitive decline, aging, dementia

The increasing proportion of the old aged population worldwide has been followed by the enhanced prevalence of Psychiatric Disorders (PD) in this age group (1, 2). Neurocognitive disorders (cognitive decline and dementia), age-related brain changes, and psychosocial issues related to aging may influence the presentation, course, and management of PD in late life (3–6). There is a need to focus efforts to innovate and improve care for the elderly with cognitive disorders and other mental health condition (7–9). This Research Topic aimed to explore the association between age-related brain changes and the long-term outcomes of PD.

Late-onset bipolar disorder is more likely to be associated with structural brain changes, depressive symptoms, and cognitive deficits (10, 11). In contrast, patients with behavioral variant frontal dementia are misdiagnosed as having bipolar disorder due to overlap in symptoms (12, 13). Similarly, Depression in late life is linked to functional and microstructural changes in the brain and is a major risk factor for dementia (14, 15). Depressive symptoms, on the other hand, are common in patients with dementia and respond relatively poorly to treatment (16). It is often challenging to delineate these diagnoses in someone presenting with both depressive symptoms and cognitive impairment. This special issue highlights some of these issues and focuses on the interplay between biological and clinical characteristics during overlapping presentations of psychiatric and cognitive disorders, their management, and their relevance to psychosocial issues such as end-of-life decision-making capacity.

The study by Aguera-Ortiz et al. aims to address the heterogeneity of clinical practice in patients with dementia and depressive symptoms. The authors conducted a multicentre, two-round Delphi survey among experts with 53 questions ranging from risk factors, signs and symptoms, and management of depression in dementia. There was an excellent consensus regarding depression being a risk factor for the onset and progression of dementia and presenting as a prodromal symptom of dementia. The authors emphasized the importance of routine cognitive examination using clinical scales and collateral information in the elderly with depression. The panel was skeptical about using the term 'pseudodementia' consistent with other literature on this topic. The panel agreed that the presentation of depression might differ based on the stage of dementia and recommended using specific criteria for depression in dementia rather than commonly used criteria for major depression. Experts recommended a trial of antidepressants in depression with dementia but agreed that antidepressants are less effective than populations with major depressive disorder without cognitive disorder. The panel recommended medications such as duloxetine and venlafaxine while recommending against tricyclic antidepressants. This study has several limitations; first, it was done in one country (Spain) and thus may be biased toward specific clinical practices in that country. Secondly, it relied on expert opinion elicited using closed-ended questions. Nevertheless, this study highlights certain controversies on this topic and the need for more research.

Other studies on this issue have explored the intricate connections between psychiatric and neurogenerative affections through different perspectives. Maia da Silva et al. reviewed the common and distinctive clinical, genetic, and neuroimaging features of Bipolar Disorder (BD) and Behavioral variant Frontotemporal Dementia (bvFTD). The paper highlights the remarkably overlapping phenomenological and biological changes shared by the disorders, which include an array of mood and cognitive abnormalities and prominent dysfunctions in frontotemporal circuitry. Also noteworthy, as described by the authors, is the occurrence of cases with typical bvFTD symptomatology that do not follow the expected pattern of functional decline. This condition, referred to as *bvFTD Phenocopy Syndrome*, could be associated with deteriorating end-stage presentations of BD. Most importantly, further investigation on this clinical entity may hint about a possible link between the pathophysiology of mood and neurodegenerative disorders.

Likewise, neuropsychological characteristics of older subjects with Attention-Deficit/Hyperactivity Disorder (ADHD) and Mild Cognitive Impairment (MCI) have been cross-sectionally assessed by Mendonca et al. After adjustment for multiple confounders, individuals with these diagnoses presented poorer performances in verbal memory and executive function tasks than controls. Interestingly, ADHD and MCI did not differ concerning cognitive profiles, suggesting that differential diagnosis between these conditions could be challenging in clinical practice. Mostly, it is intriguing to note that, despite long-term clinical and social outcomes related to ADHD (e.g., social isolation, physical inactivity, obesity, smoking, depression, and traumatic brain injury) overlap with many established risk factors for late-life cognitive impairment [see Livingston et al. (9)], data on the relationship are scarce in the literature.

Additional themes addressed in this issue concern neuropsychological aspects of Alzheimer's Disease (AD). Frankenberg et al. investigated the correlates between semantic and episodic autobiographical memory (AM) and brain metabolic rates analyzed by positron emission tomography with 18F-fluorodeoxyglucose (FDG-PET) in MCI, mild AD, and controls. Decreased patterns of cerebral activity were detected in the frontal cortex, mesial temporal substructures, and occipital cortex in MCI and AD relatively to controls. In addition, higher values in the posterior cingulum and left temporal-prefrontal areas were found in MCI compared to AD and controls, suggesting compensatory over activation in early-stage neurodegenerative disorders. Finally, alterations in semantic and episodic AM in MCI and AD correlated with increased and diminished activities in the mesial temporal substructures. Therefore, it could be inferred that AM deficits may manifest both neurodegenerative and compensatory changes within the AD spectrum (Frankenberg et al.).

Finally, Kotzé et al. assessed the end-of-life decision-making capacity (DMC) and the capacity to consent to treatment among older patients with serious mental illnesses (SMI), including schizophrenia and other psychotic disorders. Capacity to consent to treatment correlated with all domains of DCM. Using the semi-structured DMC evaluation, 65% of the participants were deemed capable, 31% were not capable, and 4% presented an uncertain degree of DMC. After controlling for age, level of education, and admission status, subjects with bipolar disorder or major depressive disorder were 4.61 times more likely to present DMC than those with psychotic disorders. Odds of having unimpaired DCM were 7.51 times higher among patients admitted voluntarily or assisted compared to involuntary cases. Finally, odds of showing preserved DCM were nearly 3-fold higher among participants with tertiary than in lower educational levels. These findings should increase awareness among healthcare providers about their duty to initiate advance care discussions, optimize decision-making capacity, and protect autonomous decision-making of older patients with SMI.

There is an emerging consensus in the field that the trajectory of PD affects the likelihood of developing dementia, and the use of advanced tools, including genetic tests, molecular and functional neuroimaging, are becoming more critical as tools in understanding the mechanisms underlying cognitive decline in this population. Despite the advances, most of the neurobiological underpinnings of PD still await further elucidation, and the diagnosis and treatment of PD and cognitive disorders at this time is primarily based on clinical history taking (Mendonca et al.), neuropsychological follow-up and a refined psychopathology exam (Maia da Silva et al.). Thus, more research is needed to further understand the interplay between specific components of the aging process including neuroplasticity and changes in specific brain networks and compensatory mechanisms, and the trajectory of psychiatric illness including the role of personality traits and genetic endophenotypes, and their impact on the onset and outcome of cognitive disorders in the elderly (17, 18).

## Author Contributions

FS and GA: literature review and writing of the manuscript. SK: literature review and compilation and writing of the manuscript. All authors contributed to the article and approved the submitted version.

## Funding

This work was supported in part by an Academic Scholars Award to SK from the Department of Psychiatry, University of Toronto. SK has also received research support from Brain and Behavior Foundation, National Institute on Aging, BrightFocus Foundation, Brain Canada, Canadian Institute of Health Research, Canadian Consortium on Neurodegeneration in Aging, Center for Aging and Brain Health Innovation, Center for Addiction and Mental Health, and equipment support from Soterix Medical.

## Conflict of Interest

The authors declare that the research was conducted in the absence of any commercial or financial relationships that could be construed as a potential conflict of interest.

## Publisher's Note

All claims expressed in this article are solely those of the authors and do not necessarily represent those of their affiliated organizations, or those of the publisher, the editors and the reviewers. Any product that may be evaluated in this article, or claim that may be made by its manufacturer, is not guaranteed or endorsed by the publisher.
